# R: IgG deposition in IgA nephropathy patients

**DOI:** 10.12861/jrip.2013.15

**Published:** 2013-06-01

**Authors:** Azar Baradaran

**Affiliations:** ^1^Department of Pathology, Isfahan University of Medical Sciences, Isfahan, Iran

**Keywords:** Immunoglobulin A nephropathy, Immune deposits

**Implication for health policy/practice/research/medical education:**

Immunostaining data in IgA nephropathy patients needs more test to investigate its potential clinical significance, especially with regards to Oxford classification.

## Dear Editor-in-Chief,

I read with great interest the recently published article by Nasri H. entitled “IgG deposition in IgA nephropathy patients” in the esteemed *Journal of Renal Injury Prevention.* In this study on 114 biopsies of IgA nephropathy (IgAN) patients they found no significant association of IgG deposits with age. There was no correlation of IgG deposit with four morphologic variables of Oxford classification (1). At this study, I would like to point out a few points. Recently, Wada *et al*. conducted a study on 57 IgAN patients who divided into two groups: IgA+IgG deposition (IgA-IgG) group and IgA deposition alone (IgA) group. They found, proteinuria was greater in the IgA-IgG group than the IgA group. Capillary wall IgA deposits were noted more frequently in the IgA-IgG group than the IgA group. They concluded that mesangial IgG deposition is associated with more severe clinical features in patients with IgAN (2). Previously Yoshimura *et al*. suggested that capillary IgA deposition is closely linked to clinical and histologic activities of IgAN and is considered to be an important factor responsible for the progression of the disease, possibly through crescent formation (3). However, this aspect of IgAN needs more test to investigate the potential clinical significance of immunostaining data, especially with regards to Oxford classification.

## Author’s contribution

AB is the single author of the manuscript.

## Conflict of interests

The authors declared no competing interests.

## Ethical considerations

Ethical issues (including plagiarism, misconduct, data fabrication, falsification, double publication or submission, redundancy) have been completely observed by the authors.

## Funding/Support

None.
